# Performance of Risk Assessment Models for Prevalent or Undiagnosed Type 2 Diabetes Mellitus in a Multi-Ethnic Population—The Helius Study

**DOI:** 10.5334/gh.846

**Published:** 2021-02-12

**Authors:** Morgan O. Obura, Irene GM van Valkengoed, Femke Rutters, Leen M. ’t Hart, Simone P. Rauh, Eric Moll van Charante, Marieke B. Snijder, Joline W.J. Beulens

**Affiliations:** 1 Amsterdam UMC, Vrije Universiteit Amsterdam, Department of Epidemiology and Biostatistics, Amsterdam Public Health research institute, Amsterdam, NL; 2 Amsterdam UMC, Academic Medical Center, Department of Public Health, Amsterdam Public Health research institute, Amsterdam, NL; 3 Department of Cell and Chemical Biology and Department of Biomedical Data Sciences, section Molecular Epidemiology, Leiden University Medical Center, Leiden, NL; 4 Amsterdam UMC, Academic Medical Center, Department of Clinical Epidemiology, Biostatistics and Bioinformatics, Amsterdam Public Health research institute, Amsterdam, NL; 5 Julius Center for Health Sciences and Primary Care, Univerisity Medical Center Utrecht, Utrecht, NL

**Keywords:** Calibration, discrimination, multi-ethnic, prediction models, risk assessment models, type 2 diabetes

## Abstract

**Background:**

Most risk assessment models for type 2 diabetes (T2DM) have been developed in Caucasians and Asians; little is known about their performance in other ethnic groups.

**Objective(s):**

We aimed to identify existing models for the risk of prevalent or undiagnosed T2DM and externally validate them in a multi-ethnic population currently living in the Netherlands.

**Methods:**

A literature search to identify risk assessment models for prevalent or undiagnosed T2DM was performed in PubMed until December 2017. We validated these models in 4,547 Dutch, 3,035 South Asian Surinamese, 4,119 African Surinamese, 2,326 Ghanaian, 3,598 Turkish, and 3,894 Moroccan origin participants from the HELIUS (Healthy LIfe in an Urban Setting) cohort study performed in Amsterdam. Model performance was assessed in terms of discrimination (C-statistic) and calibration (Hosmer-Lemeshow test). We identified 25 studies containing 29 models for prevalent or undiagnosed T2DM. C-statistics varied between 0.77–0.92 in Dutch, 0.66–0.83 in South Asian Surinamese, 0.70–0.82 in African Surinamese, 0.61–0.81 in Ghanaian, 0.69–0.86 in Turkish, and 0.69–0.87 in the Moroccan populations. The C-statistics were generally lower among the South Asian Surinamese, African Surinamese, and Ghanaian populations and highest among the Dutch. Calibration was poor (Hosmer-Lemeshow p < 0.05) for all models except one.

**Conclusions:**

Generally, risk models for prevalent or undiagnosed T2DM show moderate to good discriminatory ability in different ethnic populations living in the Netherlands, but poor calibration. Therefore, these models should be recalibrated before use in clinical practice and should be adapted to the situation of the population they are intended to be used in.

## Introduction

There is evidence that a substantial proportion of people living with T2DM may be undiagnosed [1]. Due to its asymptomatic nature at onset, T2DM diagnosis is often delayed. Consequently, by the time of diagnosis, most patients have one or more vascular complications [2]. Therefore, early detection of those with T2DM is essential for the initiation of relevant interventions, which could prevent or delay associated complications. Based on recent systematic reviews [3,4], a substantial number of risk assessment models have been developed for prevalent or undiagnosed T2DM. However, about 20% of these models have not been externally validated [5,6,7]. Evaluation of model performance in the population where the model was developed, gives optimistic results. Therefore, an external validation in an independent population is necessary to examine the model’s generalizability [8]. This may even be more urgent as most of these models were developed in Caucasian and Asian populations [3,4]. Different ethnic groups have varying risks of T2DM. For example, South Asian origin populations have a higher risk compared to Caucasian populations [9]. Furthermore, T2DM risk factors could differ by ethnic groups, hence risk factors that are informative in one ethnic group may be uninformative in another. The burden of T2DM in migrant ethnic minorities is an increasing health concern in Europe. Due to factors like socio-economic status, high-risk migrant groups are usually under-treated. This has led to higher T2DM prevalence among migrant groups than the native people [10]. Therefore, more attention for screening of high-risk individuals in these ethnic minorities is warranted. Yet, very few studies (<2%) have externally validated risk assessment models for prevalent or undiagnosed T2DM in different ethnic populations than the development population (validation C-statistics ranging from 0.59–0.80) [3]. Therefore, little is known about the performance of such models across different ethnic groups. We therefore aimed to identify existing risk assessment models for the risk of prevalent or undiagnosed T2DM and externally validate them in a large multi-ethnic cohort including validation in the ethnic subgroups represented therein.

## Methods

### Search strategy and selection

From literature, we identified existing risk assessment models for the risk of prevalent or undiagnosed T2DM and subsequently externally validated them in a large multi-ethnic cohort. We searched PubMed for relevant studies conducted in humans until December 2017. The search string is presented in Appendix A. Furthermore, we performed a reference search of the papers identified based on our search to find more relevant articles.

Articles were included if 1) the primary aim of the article was the development of a new risk assessment model for prevalent or undiagnosed T2DM (including impaired glucose regulation), 2) the risk assessment models had at least two prediction variables, 3) participants were adults, and 4) the articles were written in English. Articles were excluded if 1) they only validated an already existing risk assessment model, 2) the population was pre-selected for risk factors or disease (i.e., not the general population), and/or 3) the models included variables not present in our study and of which we could not use a proxy variable as substitute. For example, we excluded models with predictors such as monthly income and gestational diabetes because we did not have such variables and reliable proxies in our dataset.

The titles and abstracts of all articles were independently screened by one person (MO) as well as a group of reviewers (FR, IV, and MBS). The full text review for all the articles was done by one reviewer (MO) as well as a group of reviewers (FR and IV). One reviewer (MO) extracted data from all the articles, while another reviewer (IV) duplicated the data extraction from a third of the articles, randomly selected. In articles that had more than one risk assessment model, the model that was recommended by the authors was chosen. The extracted data items included the first author’s name, year of publication, country, population, population age, outcome definition, number of cases and sample size, risk predictors in the model, statistical model, measures of model performance, and whether the model was internally or externally validated (Table 1). Conflicts between reviewers were solved by review from at least one other reviewer to reach consensus (IV and MBS).

**Table 1:** Characteristics of risk assessment models for predicting risk of prevalent and/or undiagnosed T2DM.

Study	Year	Country	Ethnicity	Age (SD)/range	Definition of T2DM as reported (outcome definition)	Cases/Sample size	Risk predictors in the model	Internal validation AUC (95%CI where stated)	Statistical model	Internal (I) and external (E) validation

1. Al Khalaf *et al*.	2010	Kuwait	Caucasians	36.2 (8.9)	Diagnosis of T2DM based on ADA 2003 criteria, If FPG was ≥ 7.0 mmol/L or random glucose was ≥ 11.1 mmol/L, participants were classified as having newly diagnosed T2DM	120/560	Age, waist circumference, blood pressure medication, diabetes in sibling	0.82	Logistic	Compared with other risk scores
2. Al-Lawati *et al*.	2007	Oman	Caucasians	Age (SD) Males = 38.4 (13.7)Females = 36.7 (12.8)	T2DM was diagnosed according to 1998 WHO criteria for OGTT (FPG 11.1 mmol/l 2-h post 75-g glucose load	485/4,881	Age, waist circumference, BMI, family history of diabetes, HTN	0.83 (0.82–0.84)	Logistic	I
3. Baan *et al*.	1999	The Netherlands	Caucasians	Range: 55–75 yrs	T2DM defined as use of antidiabetic medication (insulin or oral hypoglycaemic medication) and/or 2-h PG ≥ 11.1 mmol/L according to WHO criteria	118/989	Age, sex, use of antihypertensive medication, obesity (BMI ≥ 30)	0.74 (0.70–0.78)	Logistic	E Validation: Hoorn study
4. Bang *et al*.	2009	USA	Multi-ethnic (NHANES)	58.3 (1.65) for the cases	Undiagnosed T2DM defined as FPG ≥ 7.0 mmol/L (≥126 mg/dL)	210/5,258	Age, sex, family history of diabetes, HTN, obesity (BMI or waist circumference), physical activity	0.79	Logistic	Compared with other models + E Validation: (ARIC/CHS)
5. Barengo *et al*.	2016	Colombia	Caucasians	47.2 (15.1)	ADA 2004 criteria Individuals who had fasting plasma glucose level ≥ 126 mg/dl or 2h plasma glucose ≥ 200 mg/dl were classified as having T2DM. People with T2DM, IGT or IFG were classified as having IGR	IGR = 565/2,060	IGR model: age, waist circumference, antihypertensive drug therapy and family history of diabetes (Biological father, mother or sibling)	0.72 (0.69–0.74)	Logistic	Compared their model with a validated FINDRISC model
6. Berber *et al*.	2001	Mexico	Caucasians	Age (SD) 39.0 (7.1) for men 39.1 (14.3) for women	T2DM was defined as a FPG of 7.0mmol/l and/or 2hPG 11.1mmol/l	Men 172/2,426Women 346/5,939	Men: Smoking, age, BMIWomen: WHR, BMI, age	NS, but they report the Nagelkerke, r^2^ = 0.104 for men and Nagelkerke, r^2^ = 0.031 for women	Logistic	NS
7. Chaturvedi *et al*.	2008	India	Asian	Range: 35–64 yrs	Undiagnosed T2DM defined as those with FPG ≥ 126 mg/dL (≥ 7.0 mmol/L) but who were not aware of their glycaemic status	199/4,044	Age, blood pressure, waist circumference, family history of diabetes	0.72 (0.68–0.75)	Logistic	EValidation: Data from multi-centre cross-sectional baseline survey
8. Chien *et al*.	2010	Taiwan	Asian	HbA1c < 7% (53mmol/mol) = 51.0 (10.9)HbA1c ≥ 7% (53mmol/mol) = 56.6 (10.2)	Abnormally high HbA1c levels were defined as ≥ 7% (53mmol/mol)	323/17,773	Age, sex, family history of diabetes, BMI, waist circumference, and systolic blood pressure	0.71 (0.66– 0.76)	Logistic	I, and they compared the models with the Cambridge model
9. Dong *et al*.	2011	China	Asian	54.4 (7.8)	Diagnosis of T2DM was made according to the WHO 1999 diagnostic criteria: FPG level ≥ 7.0 mmol/L or 2hPG level ≥ 11.1 mmol/L	Total sample size 2,985, cases NS	Age, BMI, WHR, systolic pressure, diastolic pressure, heart rate, and family history of diabetes (any)	0.82 (0.78–0.86)	Logistic	I
10. Dugee *et al*.	2015	Mongolia	Asian	46.4 (8.1)	Undiagnosed T2DM was defined as fasting blood glucose levels ≥ 6.1 mmol/l	59/1,018	Sex, waist circumference, HTN or medication for high blood pressure, elevated glucose, leisure time physical activity and sitting time 6 hours or more during day	0.76 (0.70–0.82)	Logistic	I
11. Gao *et al*.	2010	China	Asian	Men = 26.5 (3.5) Women = 26.1 (3.9)	T2DM defined according to 2006 WHO/IDF criteria. In individuals without known T2DM, undiagnosed T2DM was determined if person had FPG ≥ 7.0 mmol/L and/or postchallenge PG ≥ 11.1 mmol/L	Men = 81/741Women = 113/1,245	Age, waist circumference, family history of diabetes	0.64 (0.59–0.68) in men0.69 (0.64–0.72) in women	Logistic	I
12. Glümer *et al*.	2004	Denmark	Caucasians	46.0 (7.9)	Individuals without known T2DM and with FPG ≥ 7.0 mmol/L or 2-h PG ≥ 11.1 mmol/L defined as having SDM	135/3,250	Age, BMI, sex, known HTN, physical activity at leisure time, family history of diabetes	0.80 (0.77–0.84)	Logistic	I and E Validation: ADDITION pilot study
13. Gray *et al*.	2013	Portugal	Caucasians	51.5 (16.5)	Participants were classified as having IFG if their fasting glucose was ≥ 5.6 mmol/l and T2DM was defined as a fasting glucose result of ≥ 7.0 mmol/l	IFG = 338/3,374T2DM = 50/3,374	Age, sex, BMI and current HTN	For IFG/T2DM.0.70 (0.68, 0.72)	Logistic	EValidation: EPI-Porto study
14. Gray *et al*.	2012	UK	Multi-ethnic i.e. 76.5% Caucasian and 23.5 % other ethnicities (91% being south Asians)	57.3 (9.6)	IGR diagnosed using WHO 2011 diagnostic criteria and T2DM diagnosed using OGTT or HbA1c ≥ 6.5% (48 mmol/mol) For this study IGR refers to the composite of IGT and/or IFG	IGR/DM = 1,412/6,390	Age, ethnicity, sex, family history of diabetes (any type), antihypertensive therapy and BMI	0.70 (0.68, 0.72)	Logistic	EValidation: Screening Those At Risk (STAR)
15. Gray *et al*.	2010	UK	Multi-ethnic (i.e. 73% white European, 22% South Asian and others)	Aged 40–75 years 57.3 (9.6)	Participants diagnosed with T2DM according to WHO criteria with FPG ≥ 7mmol/L and/or 2-h PG ≥ 11.1 mmol/L. IFG defined as FPG between 6.1 and 6.9 mmol/L inclusive	IGR/DM = 1,249/6,186	Age, ethnicity, sex, first-degree family history of diabetes, antihypertensive therapy or history of HTN, waist circumference, BMI	0.69 (0.68–0.71)	Logistic	EValidation: Screening Those At Risk (STAR
16. Gül *et al*.	2014	US	Caucasians	For DM = 57.4 (7.7)	T2DM self-reported using a questionnaire on medical history	2,593/5,639	Familial diabetes history, high blood pressure, cholesterol, and BMI	0.77	Logistic	NS
17. Hao zhou *et al*.	2017	China	Asian	48.2 (6.8)	The cases of undiagnosed T2DM were ascertained by fasting glucose level without OGTT or HBA1c	234/5,453	Sex, age, family history of diabetes, physical activity, waist circumference, dyslipidemia, diastolic blood pressure, BMI	0.72 (0.71–0.73)	Logistic	E and compared with other scores Validation: Henan population
18. Heianza *et al*.	2013	Japan	Asian	48.4 (9.6)	The cases of undiagnosed T2DM were ascertained by fasting plasma glucose ≥ 7.0 mmol/L or glycated hemoglobin ≥ 6.5%)	965/33,335	Age, sex, family history of diabetes, current smoking habit, BMI, and HTN	0.77 (0.76–0.78)	Logistic	E and compared with existing scoresValidation: Toranomon Hospital Health Management Center
19. Keesukphan *et al*.	2007	Thailand	Asian	48.4 (10.9)	T2DM defined based on 75-g OGTT and WHO Diabetes Study Group	NS/429	Age, BMI, known HTN	0.74	Logistic	EValidation: NS clearly
20. Lee *et al*.	2012	South Korea	Asian	51.2 (0.8)	Undiagnosed T2DM was defined as a fasting plasma glucose ≥ 126 mg/dL and/orNon-fasting plasma glucose ≥ 200mg/dL	341/9,602	Age, family history of diabetes, HTN, waist circumference, smoking and alcohol intake	0.73 (0.72–0.74)	Logistic	I
21. Pires de Sousa *et al*.	2009	Brazil	Multi-ethnic	Age range 25–64yrs	FPG > 126 mg/dL (7.0 mmol/L), that is, provisional diagnosis of T2DM according to ADA criteria, classified as T2DM patients	118/1,224	Age, BMI, known HTN	0.77	Logistic	EValidation: Ouro Preto, Brazil
22. Pongchaiyakul *et al*.	2011	Thailand	Asian	47.0 (10.4) for women 49.4 (11.0) for men Mean age (range) = 48(15–85yrs)	T2DM was diagnosed using the WHO Criteria using FPG 126 mg/dl and repeated within 1 week	n = 125/1,693 for men n = 98/2,621 for women n = 223/4,314 for total population	Age, BMI and SBP for both men and women	0.75 (0.71–0.78) total population 0.70 for women 0.77 for men.	Logistic	I
23. Wang *et al*.	2013	China	Asian	53.2 (10.4)	T2DM was defined as having a fasting plasma glucose level of more than 7.0 mmol/L and/or self- reported current treatment with anti-diabetes medication (insulin or oral hypoglycemic agents)	561/6,480	Sex, family history of diabetes, physical activity, pulse pressure and waist circumference.	0.74 (0.72–0.76)	Logistic	I
24. Xie *et al*.	2010	China	Asian	35–74 years	Participants without a previous diagnosis of T2DM were categorized as follows: undiagnosed T2DM (FPG ≥ 7.0 mmol/L) and impaired fasting glycaemia (6.1 to 6.9 mmol/L). T2DM was defined as self-reported history of diabetes plus undiagnosed T2DM	994/15,540	Men: waist circumference, age, and WHR Women: WHR, waist circumference and BMI	Men = 0.71 Women = 0.73	Logistic	I
25. Zhou *et al*.	2013	China	Asian	Age range 20–74 years Men 44 (14) Women 44 (13)	Undiagnosed T2DM was detected based on fasting plasma glucose ≥ 7.0 mmol/L or 2-h plasma glucose ≥ 11.1 mmol/L	2,720/41,809	Age, sex, waist circumference, BMI, systolic blood pressure, and family history of diabetes	0.75 (0.74–0.76)	Logistic	EValidation: Two studies in Qingdao.

AUC, area under the curve; ADA, American Diabetes Association; BMI, body mass index; T2DM, type 2 diabetes mellitus; WHO, World Health Organization; FPG, fasting plasma glucose; OGTT, oral glucose tolerance test; 2hPG, two-hour 75-g post load plasma glucose level; IFG, impaired fasting glucose; IGT, impaired glucose tolerance; IGR, impaired glucose regulation; NS, not stated; WHR, waist to hip ratio; HbA1c, glycated hemoglobin; SBP, systolic blood pressure; DBP, diastolic blood pressure; SDM, screen detected diabetes; HTN, hypertension.

### Assessment of study quality

The prediction model risk of bias assessment tool (PROBAST) [11] was used to assess the risk of bias (ROB) of the included development studies of the risk assessment models. This was independently assessed by two reviewers and conflicts solved by consensus. PROBAST assesses four domains of the risk assessment models: participants selection, predictors, outcome, and analysis. The tool contains a total of 20 signaling questions across the domains. These questions address issues such as the appropriateness of predictor selection methods, evaluation and reporting of relevant performance measures, inclusion and exclusion of participants, and outcome definition, among others (Supplementary Table 1).

## HELIUS (Validation study)

The HELIUS study is a population-based, multi-ethnic cohort which has been described in detail elsewhere [12,13]. In brief, baseline data was collected between 2011–2015 and included adults (18–70 years) living in Amsterdam, the Netherlands. It included people of Dutch, Ghanaian, South Asian Surinamese, African Surinamese, Moroccan, and Turkish ethnic origin. Participants were randomly sampled, stratified by ethnic group, from the Amsterdam municipality register. The HELIUS study has been approved by the Academic Medical Center (AMC) Ethical Review Board. All participants provided written informed consent.

**Figure 1:**
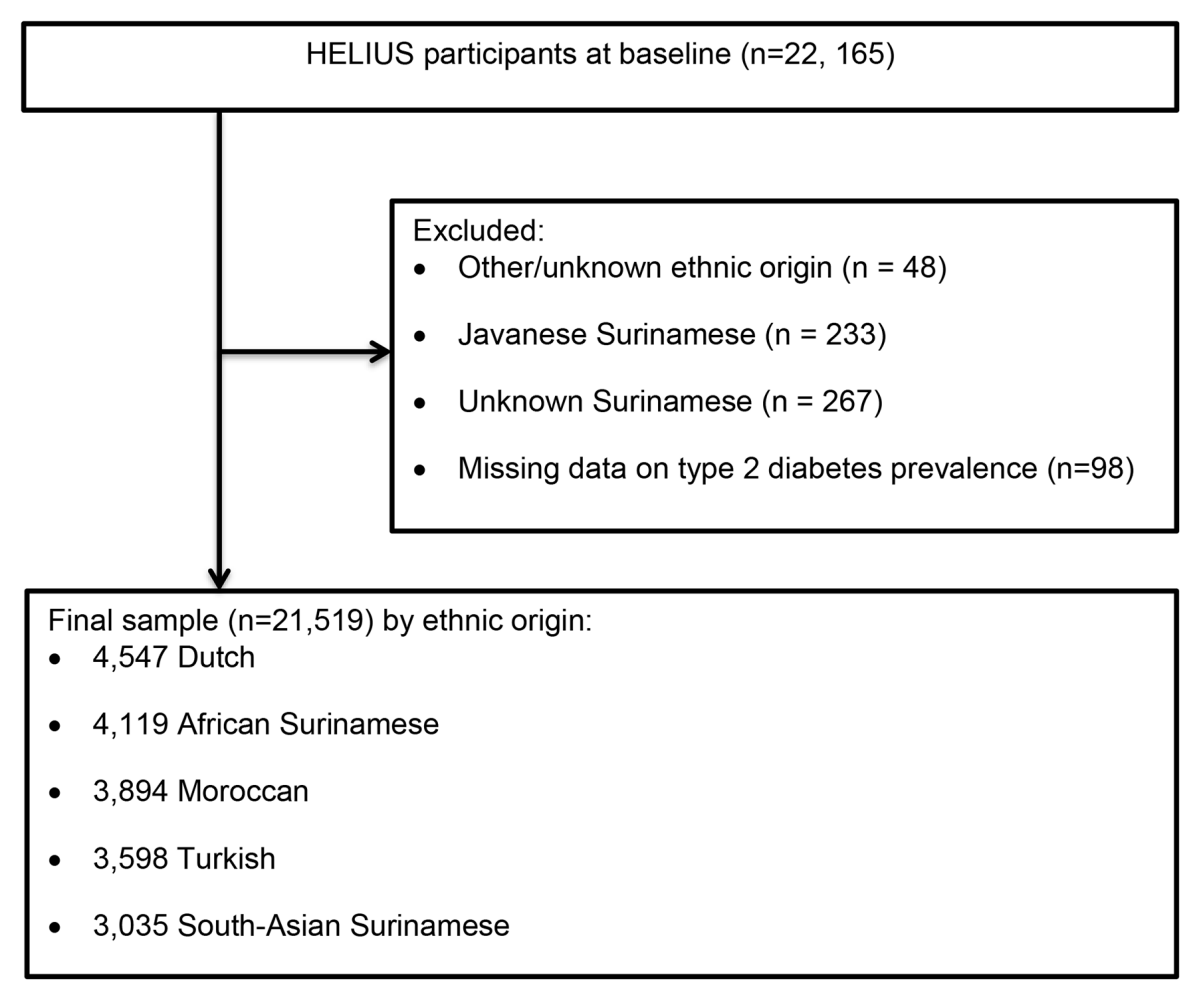
Flow chart of participants included (n = 21,519) and excluded (n = 646) in the current study.

We used baseline data of all participants in whom questionnaire data as well as data from the physical examination were available (n = 22,165). We excluded those ethnic groups with small numbers (n = 500) and those of other/unknown ethnic origin (n = 48). We additionally excluded participants who had missing data on the prevalence of T2DM (n = 98). Therefore, 21,519 participants were available for analyses, including 4,547 Dutch, 3,035 South Asian Surinamese, 4,119 African Surinamese, 2,326 Ghanaian, 3,598 Turkish, and 3,894 Moroccan participants (Figure 1).

### Predictors and other variables in HELIUS

A questionnaire was used to measure age, sex, smoking status (current/never/former), alcohol use in the past 12 months (yes/no), and family history of diabetes (yes/no/unknown). Physical activity (total minutes/week) was measured using the validated Short Questionnaire to Assess Health-Enhancing Physical Activity (SQUASH) [14], where time spent on various activities during a normal week in the past few months was recorded. Participants also brought their prescribed medications at baseline to record their medication use (e.g., use of antihypertensive drugs).

Physical examination was used to measure blood pressure (mmHg) and anthropometric measures (weight in kg, height, hip, and waist circumference in cm). Fasting blood samples were obtained to measure levels of HbA1c (mmol/mol), glucose (mmol/l), triglycerides (mmol/l), total cholesterol (mmol/l), HDL (mmol/l), and LDL (mmol/l). More information on these measurements has been described elsewhere [13].

### Ethnicity in HELIUS

Ethnicity was defined according to the participant’s country of birth as well as that of his/her parents [15]. Specifically, a participant is considered to be of non-Dutch ethnic origin if: 1) he/she was born abroad and has at least one parent born abroad (first generation) or 2) he/she was born in Netherlands but both his/her parents were born abroad (second generation).

For the Dutch sample, we invited people who were born in the Netherlands and whose parents were born in the Netherlands. After data collection, participants of Surinamese ethnic origin were further classified according to self-reported ethnic origin (obtained by questionnaire) into ‘African’, ‘South-Asian’, ‘Javanese’, or ‘other’.

### T2DM assessment in HELIUS

T2DM was considered to be present if: the participants’ fasting glucose level was ≥7.0mmol/l, and/or the HbA1c level was ≥7% (53mmol/mol), and/or the participant was using glucose-lowering medication, and/or the participant self-reported to have been diagnosed with diabetes by a health care professional. Undiagnosed T2DM was defined as having fasting glucose level ≥7.0mmol/l and/or the HbA1c level was ≥7% (53mmol/mol), in those not using glucose-lowering medication and not self-reporting a previous diagnosis of T2DM.

## Data analysis

We used the published regression coefficients and intercept values from the original models for validation. We contacted the authors to obtain the coefficients of the original model if missing. From these, we calculated the probabilities of having undiagnosed and/or prevalent T2DM in our study. We did this for the whole population and stratified by ethnic group.

We replaced the original predictor with a proxy variable if a direct match was not available in our dataset. We used family history of diabetes (i.e., if any of the parents and/or siblings has been diagnosed with diabetes) even when the model specified history of diabetes in parents only or siblings only. We used a total activity of less than 600 minutes/week as proxy for sitting time more than six hours. Total activities less than 600, 600 to 3,000, and above 3,000 minutes/week, were used as proxies for models with low, moderate, and high physical activity categories. For models that had ethnicity dichotomised as white versus others, we used the Dutch and Turkish ethnic groups as white and the other ethnic groups as others (Supplementary Table 2). Furthermore, we defined the outcome variable in our study as close as possible to the outcome definition in the development population (e.g., if the original model predicted risk of undiagnosed T2DM, we also defined our outcome as undiagnosed T2DM).

Model performance was assessed using measures of discrimination and calibration. Discrimination denotes the ability of the model to distinguish between those at high risk of having T2DM from those at low risk, while calibration indicates the ability of the model to correctly estimate the absolute risks. Discrimination was assessed using the area under the curve (AUC) also known as the C-statistic. A C-statistic of 0.5 reflects a random guess, whereas a C-statistic of 1.0 reflects perfect discrimination. We considered C-statistics between 0.6–0.7 as poor, >0.7–0.8 as moderate, >0.8–0.9 as good, and >0.9 as excellent discrimination [16]. We statistically compared the highest versus the lowest AUC across ethnic groups to ascertain if they were different across groups. This was done using the bootstrapping method (2,000 bootstraps) in the R package pROC [17].

Calibration was evaluated using the Hosmer-Lemeshow goodness of fit tests (non-significant values indicate adequate calibration). We additionally inspected the calibration plots visually. Miscalibration due to differences between the prevalence of T2DM in our study and the development populations, were ‘corrected’ for by adjusting the intercept (recalibration). This is done by adding a correction factor to the intercept of the original model, more information on this is published elsewhere [18].

Most predictors had missing values less than 1% with the exception of family history of T2DM (11.9%). Therefore, we did a multiple imputation of the missing values with 10 imputations using the multivariate imputation by chained equations (MICE) package in R.

All statistical analyses were conducted using R version 3.2.5.

## Results

**Figure 2:**
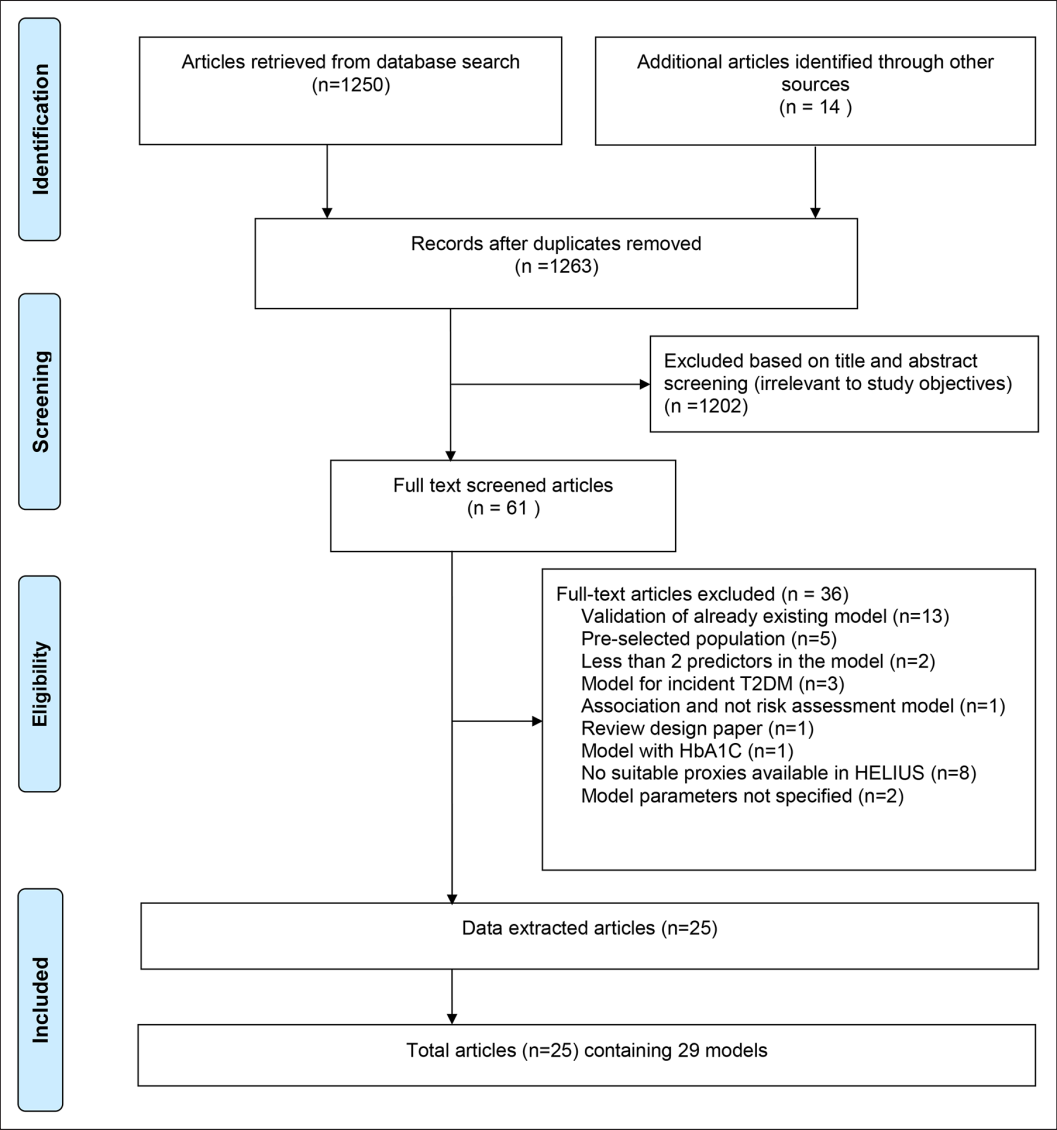
Overview of identified (n = 25) and excluded (n = 1,239) studies included in the review.

Our database search yielded 1,250 hits and 14 articles were added from the reference lists of the identified articles. After removing the duplicates (n = 1) and reviewing the titles and abstracts, 1,202 articles were excluded (Figure 2). After a full-text screening of the remaining 61 articles, 35 articles were excluded. Reasons for exclusion are provided in Supplementary File 1 (excel file attached). Twenty-six articles met our inclusion criteria, however, one of these articles had HbA1c as a predictor and was excluded because HbA1c is used for the outcome definition in our study. In total, 25 articles were included for validation (Table 1). Four articles reported risk assessment models for men and women separately [7,19,20,21], thus the current study validated a total of 29 risk assessment models from 25 articles.

Based on the four domains assessed in PROBAST, the overall ROB of the studies was generally low for the domains on predictors (100%) and outcome (97%), while it was high for most studies on the analysis domain (86%). The majority of the studies (62%) had a low ROB for the domain on participants (Supplementary Figure 1). Only 7% and 3% of the studies had unclear information to assess their ROB in the domains on participant and outcome, respectively (Supplementary Figure 1).

Thirteen studies were conducted in Asian [7,20,21,22,23,24,25,26,27,28,29,30,31], eight in Caucasian [5,19,32,33,34,35,36,37] and four in multi-ethnic [38,39,40,41] populations. However, only two [39,40] of these multi-ethnic studies reported the ethnic groups represented, and both had more than 70% Caucasians.

Included studies had different T2DM definitions: seventeen studies (69%) had models predicting risk of undiagnosed T2DM [5,19,20,21,22,24,25,26,27,28,30,31,32,33,35,38,41], four studies (15%) predicted risk of undiagnosed and impaired glucose regulation [34,36,39,40], one study predicted risk of abnormally high HbA1c values [23], one study predicted the risk of prevalent T2DM [37], and two studies (8%) developed models predicting risk of both prevalent (including undiagnosed) T2DM [7,29].

As depicted in Table 1, the internal validation AUCs ranged from 0.67–0.88 in the development populations. One study [19] did not report AUCs but only the Nagelkerke r^2^ (0.104 for men and 0.031 for women), which is the proportion of variance accounting for T2DM explained by the models. Seven studies [7,19,21,24,25,29,37] did not report an external validation of their models while three [5,23,34] only compared their models with the performance of other validated models. Sample sizes ranged from 429 [27] to 41,809 [31]. All these models included T2DM predictors that can be assessed non-invasively (i.e., without blood sampling or other invasive assessments).

The validation study included 21,519 participants (42.2% men) with a mean ± SD age of 44.3 ± 13.2 years. The age ranged from 47.9 in the African Surinamese to 40.4 in the Turkish ethnic groups while the Dutch had more men (45.8%) compared to the Ghanaians and Moroccans (38.7% for both) with the lowest number of men. T2DM prevalence varied across ethnic groups: 3.9% in Dutch, 22.2% in South Asian Surinamese, 14.4% in African Surinamese, 14.4% in Ghanaians, 11.4% in Turkish, and 12.4% Moroccan origin participants (Supplementary Table 3).

As depicted in Table 2, on average, the risk assessment models showed moderate to good discrimination, with external validation AUCs varying between 0.77–0.92 in Dutch, 0.66–0.83 in South Asian Surinamese, 0.70–0.82 in African Surinamese, 0.61–0.81 in Ghanaians, 0.69–0.86 in Turkish, and 0.69–0.87 in Moroccan ethnic groups. In general, the AUCs were consistently lowest among Ghanaians and highest among the Dutch. The highest and the lowest AUCs were statistically different (p-value < 0.05) across ethnic groups for all the models. There were no clear patterns in model performance, depending on whether the models were developed in Asian, Caucasian, or multi-ethnic populations. The four models developed separately for men and women showed that, across the ethnic groups, the AUCs for women were generally slightly higher than those for men.

**Table 2:** Performance (Areas under the Curves with 95%CIs) of 29 models for prediction of prevalent or undiagnosed T2DM, for the total population and stratified by the ethnic groups in HELIUS.


Study	Ethnic group							

	Internal validation AUC	Total population	Dutch	South Asian Surinamese	AfricanSurinamese	Ghanaian	Turkish	Moroccan

**Asian models**								
Chaturvedi *et al*. 2008	0.72	0.80 (0.79–0.81)	0.83 (0.81–0.86)	0.78 (0.76–0.80)	0.75 (0.72–0.77)	0.73 (0.69–0.76)	0.82 (0.79–0.84)	0.83 (0.82–0.85)
Chien *et al*. 2010	0.71	0.82 (0.81–0.83)	0.86 (0.81–0.91)	0.80 (0.78–0.82)	0.79 (0.76–0.81)	0.76 (0.72–0.80)	0.86 (0.83–0.88)	0.83 (0.81–0.86)
Dong *et al*. 2011	0.82	0.88 (0.87–0.88)	0.91 (0.89–0.94)	0.89 (0.87–0.90)	0.89 (0.87–0.91)	0.84 (0.81–0.88)	0.92 (0.90–0.94)	0.93 (0.92–0.94)
Dugee *et al*. 2015	0.76	0.81 (0.80–0.82)	0.81 (0.79–0.84)	0.83 (0.81–0.84)	0.79 (0.77–0.81)	0.76 (0.73–0.79)	0.82 (0.80–0.84)	0.82 (0.80–0.84)
Gao *et al*. 2010								
men	0.64	0.80 (0.79–0.82)	0.85 (0.81–0.88)	0.79 (0.76–0.82)	0.78 (0.74–0.82)	0.72 (0.67–0.78)	0.82 (0.78–0.85)	0.81 (0.78–0.85)
women	0.69	0.85 (0.84–0.86)	0.92 (0.89–0.96)	0.80 (0.77–0.83)	0.82 (0.79–0.85)	0.82 (0.78–0.86)	0.86 (0.83–0.88)	0.87 (0.85–0.89)
Hao zhou *et al*. 2017	0.72	0.82 (0.81–0.83)	0.87 (0.84–0.89)	0.79 (0.77–0.81)	0.79 (0.77–0.81)	0.74 (0.70–0.78)	0.83 (0.81–0.85)	0.83 (0.81–0.85)
Heianza *et al*. 2013	0.77	0.79 (0.78–0.80)	0.85 (0.82–0.87)	0.78 (0.76–0.81)	0.74 (0.72–0.77)	0.72 (0.68–0.76)	0.79 (0.76–0.81)	0.83 (0.81–0.85)
Keesukphan *et al*. 2007	0.74	0.76 (0.75–0.77)	0.81 (0.77–0.84)	0.74 (0.72–0.77)	0.73 (0.70–0.76)	0.70 (0.65–0.74)	0.77 (0.74–0.80)	0.77 (0.75–0.80)
Lee *et al*. 2012	0.73	0.75 (0.74–0.76)	0.79 (0.76–0.82)	0.73 (0.71–0.75)	0.71 (0.69–0.74)	0.71 (0.67–0.75)	0.75 (0.72–0.77)	0.77 (0.75–0.79)
Pongchaiyakul *et al*. 2011								
men	0.70	0.74 (0.72–0.75)	0.81 (0.77–0.85)	0.72 (0.68–0.75)	0.73 (0.69–0.77)	0.67 (0.61–0.74)	0.75 (0.71–0.79)	0.76 (0.73–0.80)
women	0.77	0.78 (0.76–0.79)	0.83 (0.78–0.89)	0.75 (0.72–0.79)	0.73 (0.70–0.77)	0.70 (0.64–0.76)	0.82 (0.79–0.85)	0.79 (0.77–0.82)
Wang *et al*. 2013	0.74	0.76 (0.75–0.77)	0.84 (0.81–0.86)	0.73 (0.71–0.75)	0.76 (0.74–0.78)	0.71 (0.68–0.73)	0.78 (0.76–0.80)	0.78 (0.76–0.80)
Xie *et al*. 2010								
men	0.71	0.73 (0.71–0.74)	0.80 (0.76–0.84)	0.70 (0.66–0.73)	0.75 (0.70–0.79)	0.70 (0.64–0.77)	0.73 (0.69–0.77)	0.73 (0.69–0.77)
women	0.73	0.81 (0.79–0.82)	0.88 (0.84–0.93)	0.76 (0.73–0.79)	0.76 (0.72–0.79)	0.72 (0.66–0.77)	0.82 (0.79–0.85)	0.84 (0.82–0.87)
Zhou *et al*. 2013	0.75	0.80 (0.79–0.81)	0.88 (0.86–0.91)	0.81 (0.79–0.83)	0.80 (0.77–0.82)	0.76 (0.72–0.79)	0.85 (0.83–0.87)	0.86 (0.84–0.87)
**Caucasian models**								
Al Khalaf *et al*. 2010	0.82	0.81 (0.80–0.82)	0.85 (0.82–0.88)	0.78 (0.76–0.80)	0.78 (0.76–0.81)	0.76 (0.72–0.80)	0.83 (0.80–0.85)	0.81 (0.79–0.83)
Al-Lawati *et al*. 2007	0.83	0.81 (0.80–0.82)	0.85 (0.82–0.88)	0.78 (0.76–0.80)	0.77 (0.74–0.79)	0.74 (0.72–0.79)	0.81 (0.79–0.84)	0.83 (0.80–0.85)
Baan *et al*. 1999	0.74	0.82 (0.81–0.83)	0.87 (0.85–0.89)	0.82 (0.81–0.84)	0.81 (0.79–0.82)	0.75 (0.72–0.78)	0.83 (0.81–0.85)	0.86 (0.84–0.87)
Barengo *et al*. 2016	0.72	0.76 (0.75–0.77)	0.79 (0.77–0.80)	0.75 (0.74–0.77)	0.75 (0.73–0.76)	0.70 (0.68–0.72)	0.76 (0.74–0.78)	0.78 (0.77–0.80)
Berber *et al*. 2001								
men	NS	0.77 (0.75–0.78)	0.82 (0.78–0.86)	0.78 (0.76–0.81)	0.73 (0.69–0.78)	0.65 (0.58–0.71)	0.81 (0.77–0.84)	0.82 (0.79–0.85)
women	NS	0.82 (0.81–0.83)	0.90 (0.85–0.94)	0.79 (0.76–0.82)	0.77 (0.74–0.80)	0.74 (0.68–0.79)	0.86 (0.83–0.88)	0.86 (0.84–0.88)
Glümer *et al*. 2004	0.80	0.82 (0.81–0.83)	0.86 (0.83–0.89)	0.81 (0.79–0.83)	0.78 (0.76–0.81)	0.74 (0.70–0.78)	0.84 (0.82–0.86)	0.85 (0.84–0.87)
Gray *et al*. 2013	0.70	0.78 (0.77–0.78)	0.81 (0.79–0.82)	0.77 (0.76–0.79)	0.75 (0.74–0.77)	0.71 (0.69–0.73)	0.80 (0.79–0.82)	0.81 (0.80–0.83)
Gül *et al*. 2014	0.77	0.67 (0.66–0.68)	0.77 (0.73–0.81)	0.66 (0.64–0.68)	0.70 (0.67–0.72)	0.61 (0.57–0.65)	0.69 (0.67–0.72)	0.69 (0.67–0.72)
**Multi-ethnic models**								
Bang *et al*. 2009	0.79	0.82 (0.81–0.83)	0.86 (0.84–0.89)	0.80 (0.77–0.82)	0.78 (0.75–0.80)	0.77 (0.73–0.80)	0.83 (0.81–0.85)	0.84 (0.82–0.86)
Gray *et al*. 2010	0.69	0.73 (0.72–0.74)	0.79 (0.77–0.80)	0.75 (0.74–0.77)	0.75 (0.74–0.77)	0.69 (0.67–0.72)	0.77 (0.75–0.78)	0.78 (0.77–0.80)
Gray *et al*. 2012	0.70	0.75 (0.74–0.76)	0.80 (0.78–0.81)	0.78 (0.77–0.80)	0.77 (0.76–0.79)	0.71 (0.70–0.73)	0.79 (0.78–0.81)	0.81 (0.80–0.83)
Pires de sousa *et al*. 2009	0.77	0.79 (0.78–0.80)	0.85 (0.82–0.88)	0.78 (0.76–0.80)	0.76 (0.73–0.78)	0.70 (0.65–0.74)	0.81 (0.79–0.84)	0.82 (0.80–0.84)

NS means Not stated.

There were no clear patterns in model performance, depending on whether the models were developed in Asian, Caucasian, or multi-ethnic populations.

Calibration was poor for all models (Hosmer-Lemeshow p < 0.001) except one [26], but only in the Dutch (Hosmer-Lemeshow p-value = 0.39). Generally, from the calibration plots, the majority of the models overestimated T2DM in all ethnic groups, especially at higher predicted risks. However, calibration improved after recalibration, see for example Lawati et al. calibration plots (Supplementary Figure 2). The recalibrated models had lower Hosmer-Lemeshow statistics, although the tests were still statistically significant (p < 0.05). Since the ranking of the predicted risks is not affected by recalibration, discrimination did not change.

## Discussion

An external evaluation of the performance of 29 non-invasive risk assessment models for prevalent or undiagnosed T2DM in a multi-ethnic cohort showed that the models had a moderate to good discriminatory ability per ethnic group. However, the AUCs were heterogeneous across ethnic populations, with the AUCs being consistently lowest among the Ghanaians and highest among the Dutch, compared to other ethnic groups. Furthermore, no clear patterns in performance of the models were witnessed depending on whether the models were developed in Asian, Caucasian, or multi-ethnic populations. The models showed poor calibration with most overestimating the predicted probabilities which improved, to some extent but not adequately, after recalibrating the models.

Our study had several strengths. All models were identified through a literature search. Our validation sample was large with participants from six ethnic populations, thereby enabling us to carry out validation stratified by ethnic groups. We additionally tried to define the outcome variable as close as possible to the outcome definition in the development population. Finally, we included undiagnosed T2DM cases, therefore reducing the false negative cases which might arise when using only verified T2DM cases. Although, undiagnosed T2DM ascertainment was partly based on a single fasting plasma glucose, which might lead to some false positives. Limitations of our study include the lack of certain predictors, nevertheless, we attempted to use available predictors, as close as possible to the missing predictors, as proxy where applicable. We also did not have a suitable proxy or data on gestational diabetes which is an important risk factor in women. The two-hour glucose was not used to ascertain T2DM in our study and thus we might have underestimated T2DM cases. Finally, we only did validation in the ethnic groups living in Amsterdam which may affect our results generalizability to similar ethnic groups living in their original countries.

Our study is unprecedented, hence, no direct comparisons can be made because there is no review, to the best of our knowledge, which has externally validated risk assessment models for prevalent or undiagnosed T2DM in a multi-ethnic population. However, there have been recent systematic reviews comparing different models for prevalent and or undiagnosed T2DM [3,4]. Unfortunately, only about 2% of these models have been externally validated in different ethnic populations than the development population, with a moderate performance on average (validation AUCs ranging from 0.59–0.80) [3]. Another study assessed the prevalence of known and newly detected T2DM, developed a risk model, and validated it in Hindustani Surinamese, African Surinamese, and Dutch ethnic groups in the Netherlands [42]. The Hindustani Surinamese had the highest T2DM prevalence while the Dutch had the lowest. The model performed moderately well with AUCs ranging from 0.74–0.80, but no calibration results were reported. All these results are somewhat congruent with our results.

Another study by Gray et al. [39], developed a risk assessment model for undiagnosed T2DM in a multi-ethnic cohort with 24% non-Caucasians. This model was externally validated in a multi-ethnic (26.3% non-Caucasians) study called STAR (Screening Those At Risk) [39]. The same model performed similarly in our study with an AUC of 0.75 in the total population and a range of 0.71–0.81 in the different ethnic groups. However, this and similar models are validated in populations with limited ethnic variation consisting predominantly of Caucasians and Asians. Therefore, due to lack of studies stratifying for other different ethnic groups, we cannot directly compare our results for the different ethnic groups.

Generally, it is expected that a risk assessment model would perform worse in an independent external validation dataset than the development dataset. Nevertheless, most models had moderate discrimination with some having better AUCs in our study than in their development study. This could be explained by the differences in heterogeneity between our study and theirs [43]. Larger heterogeneity in the validation study might lead to higher AUCs than the development population.

The majority of the models overestimated T2DM risk, especially in those with higher observed risk in some ethnic groups. This could partly be explained by poor calibration of the model in the development dataset and differences such as the prevalence of T2DM, how the outcome and predictors were measured between the validation and the development populations. These differences might influence predictions during validation thereby affecting calibration [43]. Although, we ‘corrected’ for the difference in T2DM prevalence by adjusting the intercept of the models which consequently improved calibration, but not sufficiently. The disagreement between predicted and observed risks that remained even after intercept adjustment could be because of the sensitivity of the Hosmer-Lemeshow test to sample size. With a large sample size, small differences between predicted and observed risks are likely to be considered significant even though good calibration from visually inspecting the calibration plot is evident.

However, overestimation of T2DM risk in those with higher observed risk might not directly affect public health strategies like screening. Certain thresholds for absolute disease risk are usually set before initiation of public health strategies. Therefore, overestimation of risk beyond this threshold might not cause a change in the intervention strategies. However, some models overestimated T2DM risk in those with lower observed risk. These predictions could well be under the thresholds for intervention initiation, therefore such models should be calibrated before use in clinical practice.

Performance was consistently low in the South Asian Surinamese, African Surinamese, and the Ghanaian populations compared to the other ethnic groups. This could partly be explained by the fact that risk factors for T2DM may differ across ethnic groups and it is plausible that some of the risk factors that are very informative in these populations were not captured in any of the models. Informative risk factors such as socio-economic status (e.g., education level or income) and access to healthcare vary substantially between ethnic groups. Unfortunately, none of the models that we validated had these variables. Therefore, validating a model with only the original risk factors within another population is likely suboptimal. Moreover, the models showed similar performance in the African Surinamese and Ghanaian populations, and likewise, in the Turkish and Moroccan populations. This suggests that, perhaps, performance is comparable in ethnic groups having more or less similar cultural beliefs and practices like religion, diet choice, cooking habits, etc. These factors might have an influence on the predictors of T2DM risk and how they are measured across ethnic groups. For example, assessing alcohol use in ethnic groups predominant with believers in a religion that prohibits alcohol use, might lead to ‘socially desirable’ responses. These potential differences in the way predictors are measured across ethnic groups could also in part explain the heterogeneity in model performance.

We expected the validity to be better when evaluating models developed in a similar ethnic group as the validation ethnic group. Nevertheless, there were no clear patterns in this regard, which could be due to the fact that generally the models were not ethnic specific, hence not directly analogous to the ethnic groups in our study. Additionally, some of the models were not developed in ethnic populations living in a different country than their country of origin. Studies have shown that migrant ethnic groups might be affected by underlying factors related to their new host country and country of origin, and these factors influence their risk for T2DM. Similar studies have also shown that those with migrant status have a higher risk for T2DM [10], so validating models developed in ethnic populations living in their country of origin in populations with ethnic groups with migrant status might explain the lack of clear patterns. This is perhaps strengthened by the good performance of the models in the Dutch population without a migrant status.

Model performance, in terms of discrimination, was generally better across the ethnic groups in women compared to men for the studies that developed models separately for both sexes. These results are in line with a validation study of prediction models for T2DM in a European population that also showed higher performance in women than men [44]. There is evidence to suggest that the pathogenesis of T2DM may be different between men and women. Men, compared to women, usually have higher fasting glucose levels and a lower BMI before the onset of T2DM [45,46]. Previous studies have also shown that several factors predicting T2DM differ between sexes (e.g., family history of diabetes) [47,48], possibly explaining, in part, the difference in performance for some of these models. Therefore, risk assessment models for T2DM should likely be formulated separately for men and women.

The models’ discrimination was heterogeneous and calibration was poor, therefore, external validation, including recalibration, is necessary before use in clinical practice. We additionally recommend that such models should be adapted to the situation of the population they are intended to be used in and more such models developed or improved for certain ethnic subgroups (e.g., South Asian Surinamese, African Surinamese, and Ghanaian populations).

In conclusion, existing risk assessment models for prevalent or undiagnosed T2DM showed moderate to good discriminatory ability in different ethnic populations living in the Netherlands. However, these models had poor calibration with most of them overestimating the predicted probabilities for T2DM. Furthermore, these models show heterogeneous discrimination per ethnic group with consistently lower performance in the South Asian Surinamese, African Surinamese, and Ghanaian populations, hence the need for external validation of such models in different ethnic groups and possibly an appropriate adaptation to the local setting before clinical use.

## Data Accessibility Statement

Restrictions apply to the availability of data generated or analyzed during this study to preserve patient confidentiality or because they were used under license. The corresponding author will on request detail the restrictions and any conditions under which access to some data may be provided.

## Additonal Files

The additional files for this article can be found as follows:

10.5334/gh.846.s1 Appendix A. Search strategy and search strings.

10.5334/gh.846.s2 Supplemental File1. Risk of bias assessment guidelines and results, predictors and their proxies used, study sample characteristics and calibration plots for Lawati.
