# Supplementary material for: Performance of Risk Assessment Models for Prevalent or Undiagnosed Type 2 Diabetes Mellitus in a Multi-Ethnic Population—The Helius Study
Source: Glob Heart. 2021 Feb 12;16(1):13. doi: 10.5334/gh.846 (PMC7880001; doi:10.5334/gh.846)
Supplement: Appendix A. — Search strategy and search strings. [file gh-16-1-846-s1.pdf]

Appendix A. Search strategy and search strings

References for this review were identified by searching in PubMed for studies conducted in humans and published until December 31, 2017. We used the terms in the search string below. Additional articles were identified from reference lists of identified articles.

(((("Diabetes Mellitus, Type 2"[Mesh] OR ((diabetes[tiab] OR diabetic*[tiab]) AND (non insulin depend*[tiab] OR noninsulin depend*[tiab] OR noninsulindepend*[tiab] OR non insulindepend*[tiab] OR maturity onset*[tiab] OR adult onset*[tiab] OR slow onset*[tiab])) OR dm2[tiab] OR niddm[tiab] OR dm 2[tiab] OR t2d[tiab] OR dm type 2[tiab] OR type 2 diabet*[tiab] OR dm type II[tiab] OR type two diabet*[tiab] OR type II diabet*[tiab] OR dm type II[tiab]) AND ("Prevalence"[Mesh] OR prevalen*[tiab] OR undiagnosed[tiab] OR un-diagnosed[tiab] OR "not diagnosed"[tiab] OR "non diagnosed"[tiab] OR nondiagnosed[tiab]) AND ("Risk Assessment"[Mesh] OR "Risk Factors"[Mesh] OR "Regression Analysis"[Mesh] OR "Predictive Value of Tests"[Mesh] OR risk prediction*[tiab] OR predictive model*[tiab] OR predictive equation*[tiab] OR prediction model*[tiab] OR risk calculator*[tiab] OR prediction rule*[tiab] OR risk model*[tiab] OR statistical model*[tiab] OR risk assessment*[tiab] OR risk factor*[tiab] OR predictive value*[tiab] OR risk scor*[tiab]) AND (model*[tiab])) NOT ("Animals"[Mesh] NOT "Humans"[Mesh])) NOT ("Meta-Analysis"[Publication Type] OR "Review"[Publication Type] OR "Letter"[Publication Type] OR "Editorial"[Publication Type] OR "News"[Publication Type] OR "Bibliography"[Publication Type]))
