# Supplementary material for: Performance of Risk Assessment Models for Prevalent or Undiagnosed Type 2 Diabetes Mellitus in a Multi-Ethnic Population—The Helius Study
Source: Glob Heart. 2021 Feb 12;16(1):13. doi: 10.5334/gh.846 (PMC7880001; doi:10.5334/gh.846)
Supplement: Supplemental File1. — Risk of bias assessment guidelines and results, predictors and their proxies used, study sample characteristics and calibration plots for Lawati. [file gh-16-1-846-s2.pdf]

Supplementary Table 1: Risk of bias assessment guidelines

Risk of bias domain	Low risk of bias	High risk of bias
Participants	Appropriate source of data e.g. cross-sectional/Cohort/RCT; appropriate inclusion/exclusion of participants	Inappropriate source of data; e.g. from Registry; exclusion of specific subgroups
Outcome(s)	Clear (pre-specified) definition of the outcome; outcome defined and determined similarly for all participants; predictors excluded from the outcome definition; outcome determined without knowledge of predictor information; appropriate time interval between predictor assessment and outcome determination.	Non pre-specified definition of the outcome; outcome defined and determined differently for all participants; predictors not excluded from the outcome definition; outcome determined with knowledge of predictor information; inappropriate time interval between predictor assessment and outcome determination.
Predictors	Predictors defined and assessed in a similar way for all participants; predictor assessments made without knowledge of outcome data; all predictors available at the time the model is intended to be used.	Predictors not defined and assessed in a similar way for all participants; predictor assessments made with knowledge of outcome data; all predictors not available at the time the model is intended to be used.
Analysis	Reasonable number of participants with the outcome; continuous and categorical predictors handled appropriately; appropriate handling of participants with missing data e.g. multiple imputation; selection of predictors not based on univariable analysis; complexities in the data accounted for appropriately; relevant model performance measures e.g. calibration and discrimination evaluated appropriately; model overfitting and optimism in model performance accounted for and the predictors and their assigned weights in the final model correspond to the results from multivariable analysis.	Low number of participants with the outcome; continuous and categorical predictors handled inappropriately e.g. unnecessary categorization of continuous predictors; inappropriate handling of participants with missing data e.g. Complete case analysis; selection of predictors based on univariable analysis; complexities in the data not accounted for; relevant model performance measures e.g. calibration and/or discrimination not evaluated; model overfitting and optimism in model performance not accounted for and/or the predictors and their assigned weights in the final model do not correspond to the results from multivariable analysis.

Supplementary Table 2. Predictors in the development model and their Proxies in the HELIUS study.

Study	Year	Predictors in the development model missing in the validation study	Predictors as Proxies in validation population
1. Al Khalaf et al.	2010	Diabetes in sibling	Family history of diabetes, i.e. diabetes in parents and/or siblings
2. Dugee et al.	2015	Sitting time 6 hours or more during day	Total activity of less than 600 minutes/week
3. Gray et al.	2012	Ethnicity (white European or other)	White European (Dutch and Turkish combined) and the other ethnic groups as others.
4. Gray et al.	2010	Ethnicity (white European or other)	White European (Dutch and Turkish combined) and the other ethnic groups as others.
5. Wang et al.	2013	Physical activity	Low=less than 600, moderate=600 to 3000<br>High=above 3000 minutes/week.
6. Zhou et al.	2013	First degree relatives' family history of diabetes (including offspring)	family history of diabetes, i.e. diabetes in parents and/or siblings

SUPPLEMENTARY TABLE 3 Characteristics of the study sample, for the total population and stratified by ethnic groups in the HELIUS study[1]

	Ethnic group						
	Total	Dutch	South Asian Surinamese	African Surinamese	Ghanaian	Turkish	Moroccan
N (%)	21519 (100%)	4547 (21.1%)	3035 (14.1%)	4119 (19.1%)	2326 (10.8%)	3598 (16.7%)	3894 (18.1%)
Age (years)	44.3 (13.2)	46.2 (14.0)	45.5 (13.4)	47.9 (12.5)	44.8 (11.2)	40.4 (12.2)	40.5 (12.9)
Sex, males [n (%)]	9089 (42.2%)	2082 (45.8%)	1367 (45.0%)	1602 (38.9%)	901 (38.7%)	1628 (45.2%)	1509 (38.7%)
BMI (kg/m^2)	27.1 (5.3)	24.8 (4.2)	26.3 (4.8)	27.8 (5.5)	28.5 (5.0)	28.6 (5.7)	27.6 (5.2)
Waist circumference (cm)	92.4 (13.5)	89.3 (12.9)	91.8 (13.1)	93.0 (14.0)	93.2 (12.4)	94.5 (14.1)	93.1 (13.5)
Currently smoking [n (%)]	5135 (23.8%)	1121 (24.7%)	857 (28.3%)	1297 (31.6%)	104 (4.5%)	1236 (34.6%)	520 (13.4%)
Alcohol use; *Never* [n (%)]	8815 (41.0%)	297 (6.5%)	1070 (35.4%)	990 (24.2%)	801 (34.8%)	2400 (67.2%)	3257 (84.2%)
Physical activity (minutes/week)	2507 (1731.2)	2276 (1669.4)	2594 (1735.7)	2765 (1851.2)	2501 (2132.1)	2124 (1697.9)	2276 (1669.4)
Systolic blood pressure (mmHg)	126.9 (17.7)	128.4 (18.3)	128.4 (18.3)	131.6 (18.2)	135.6 (18.9)	123.3 (15.8)	121.9 (15.9)
Family history of diabetes; Yes [n (%)]	7911 (46.9%)	793 (21.1%)	1768 (69.3%)	1581 (51.1%)	419 (28.8%)	1475 (51.4%)	1875 (59.6%)
**Biochemical Measures**							
HbA1c (mmol/mol)	39.0 (8.7)	39.1(4.9)	42.2 (10.2)	40.2 (9.5)	39.5 (9.5)	38.7 (8.6)	38.7 (8.6)
Glucose (mmol/l)	5.5 (1.2)	5.3 (0.8)	5.8 (1.5)	5.5 (1.3)	5.4 (1.2)	5.5 (1.2)	5.5 (1.3)
Triglycerides (mmol/l)	1.0 (0.7)	1.0 (0.7)	1.2 (0.8)	0.9 (0.5)	0.7 (0.4)	1.2 (0.9)	1.0 (0.6)
Cholesterol (mmol/l)	4.9 (1.0)	5.1 (1.1)	5.0 (1.0)	4.9 (1.0)	4.9 (1.0)	4.9 (1.0)	4.6 (0.9)
LDL (mmol/l)	3.0 (0.9)	3.1 (1.0)	3.1 (0.9)	3.0 (0.9)	3.0 (0.9)	3.0 (0.9)	2.8 (0.8)
HDL (mmol/l)	1.4 (0.4)	1.6 (0.5)	1.3 (0.4)	1.5 (0.4)	1.6 (0.4)	1.3 (0.4)	1.3 (0.3)
Type 2 diabetes prevalence [n(%)]	2673 (12.4%)	179 (3.9%)	675 (22.2%)	593 (14.4%)	334 (14.4%)	410 (11.4%)	482 (12.4%)
Undiagnosed type 2 diabetes [n(%)]	1387 (6.4%)	126 (2.8%)	349 (11.5%)	286 (7.0%)	121 (5.2%)	217 (6.0%)	288 (7.4%)

[1]Values are mean ± standard deviation unless stated otherwise.

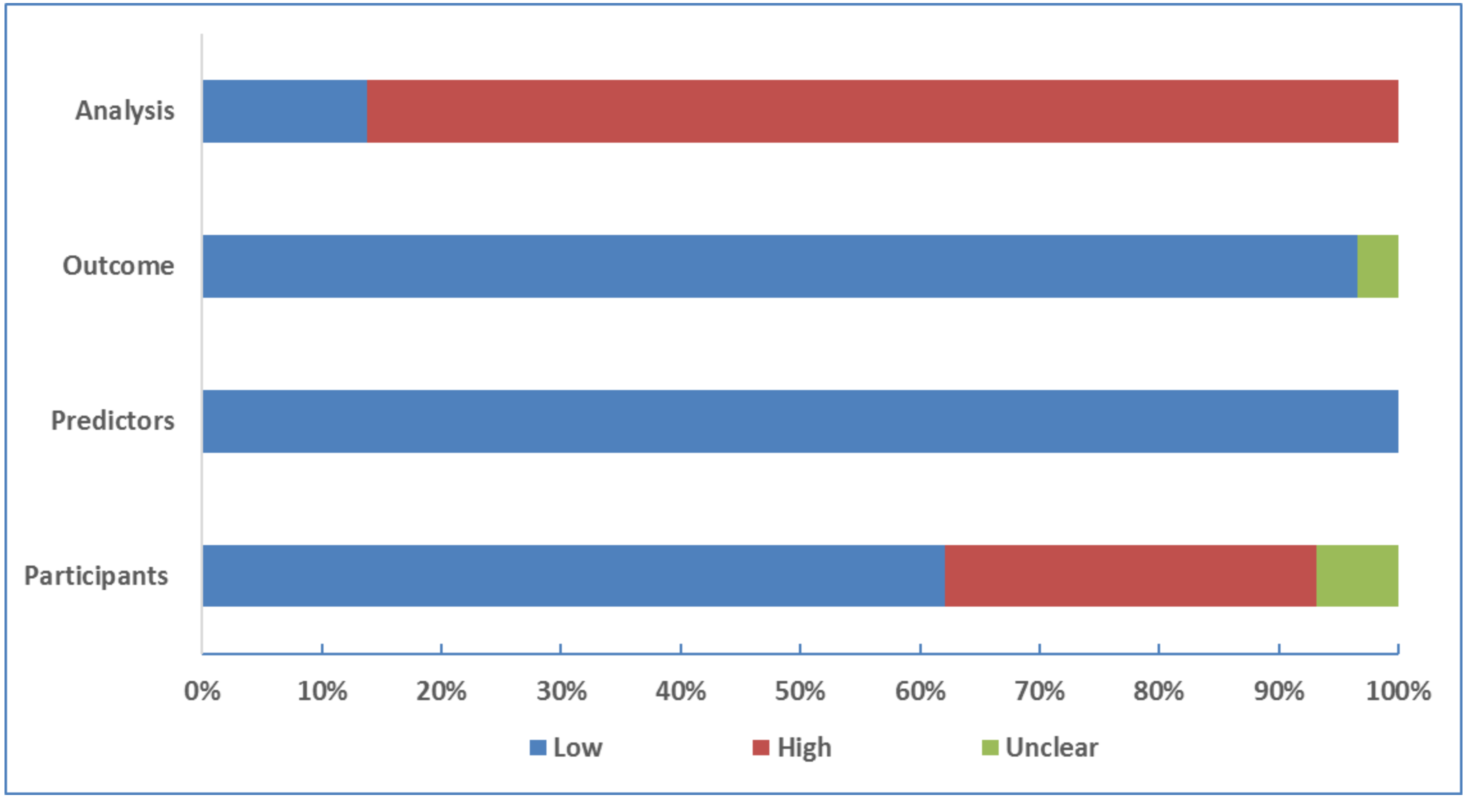

Supplementary fig 1: Risk of bias assessment per PROBAST (Prediction model Risk Of Bias ASsessment Tool) domain of included studies.

A.

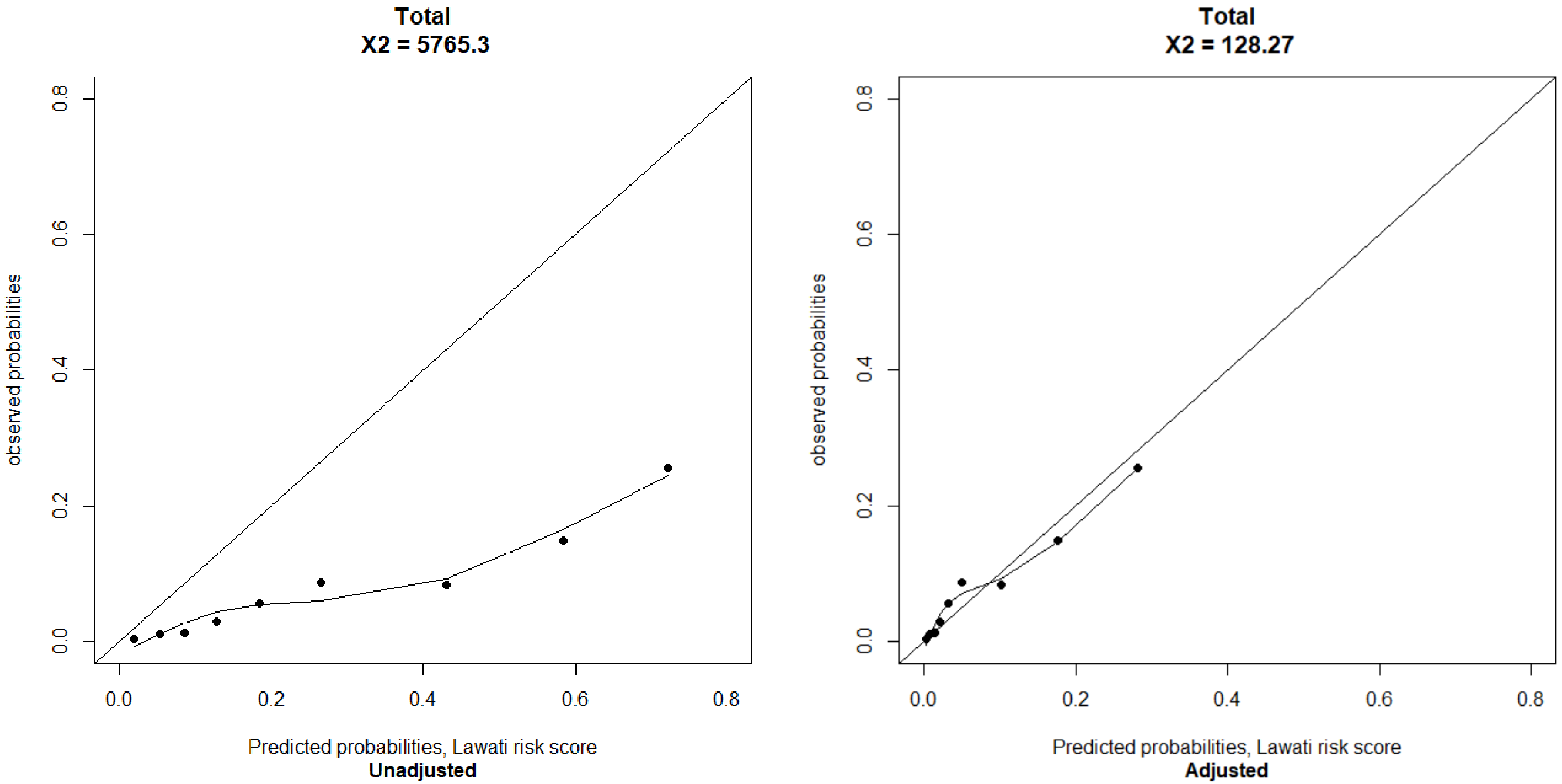
Total
X2 = 5765.3
observed probabilities
0.0
0.2
0.4
0.6
0.8
Predicted probabilities, Lawati risk score
Unadjusted
Total
X2 = 128.27
observed probabilities
Predicted probabilities, Lawati risk score
Adjusted

B.

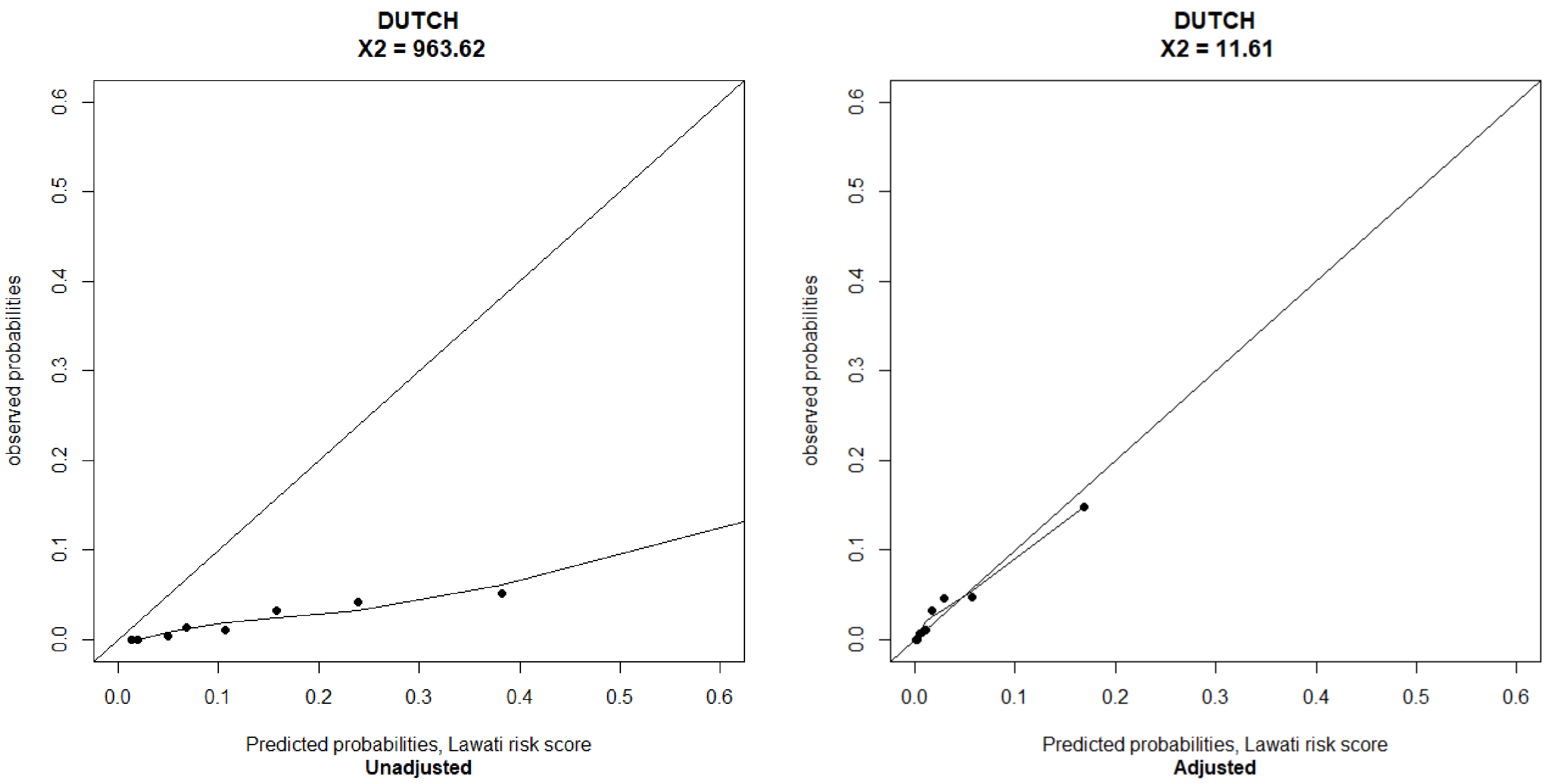
DUTCH
X2 = 963.62
observed probabilities
0.0
0.1
0.2
0.3
0.4
0.5
0.6
Predicted probabilities, Lawati risk score
Unadjusted
DUTCH
X2 = 11.61
observed probabilities
Predicted probabilities, Lawati risk score
Adjusted

C.

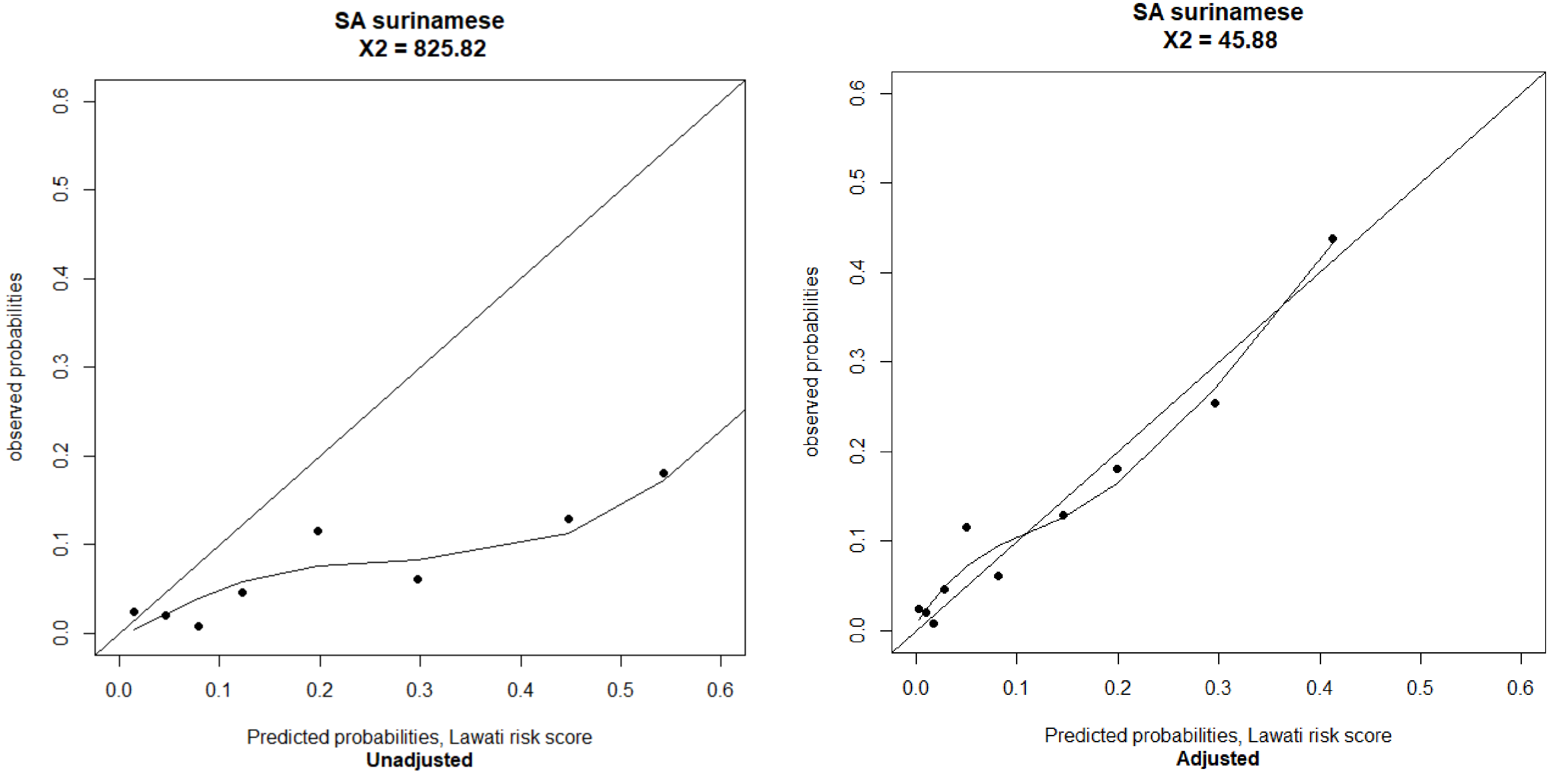
SA surinamese
X2 = 825.82
observed probabilities
Predicted probabilities, Lawati risk score
Unadjusted
SA surinamese
X2 = 45.88
observed probabilities
Predicted probabilities, Lawati risk score
Adjusted

D.

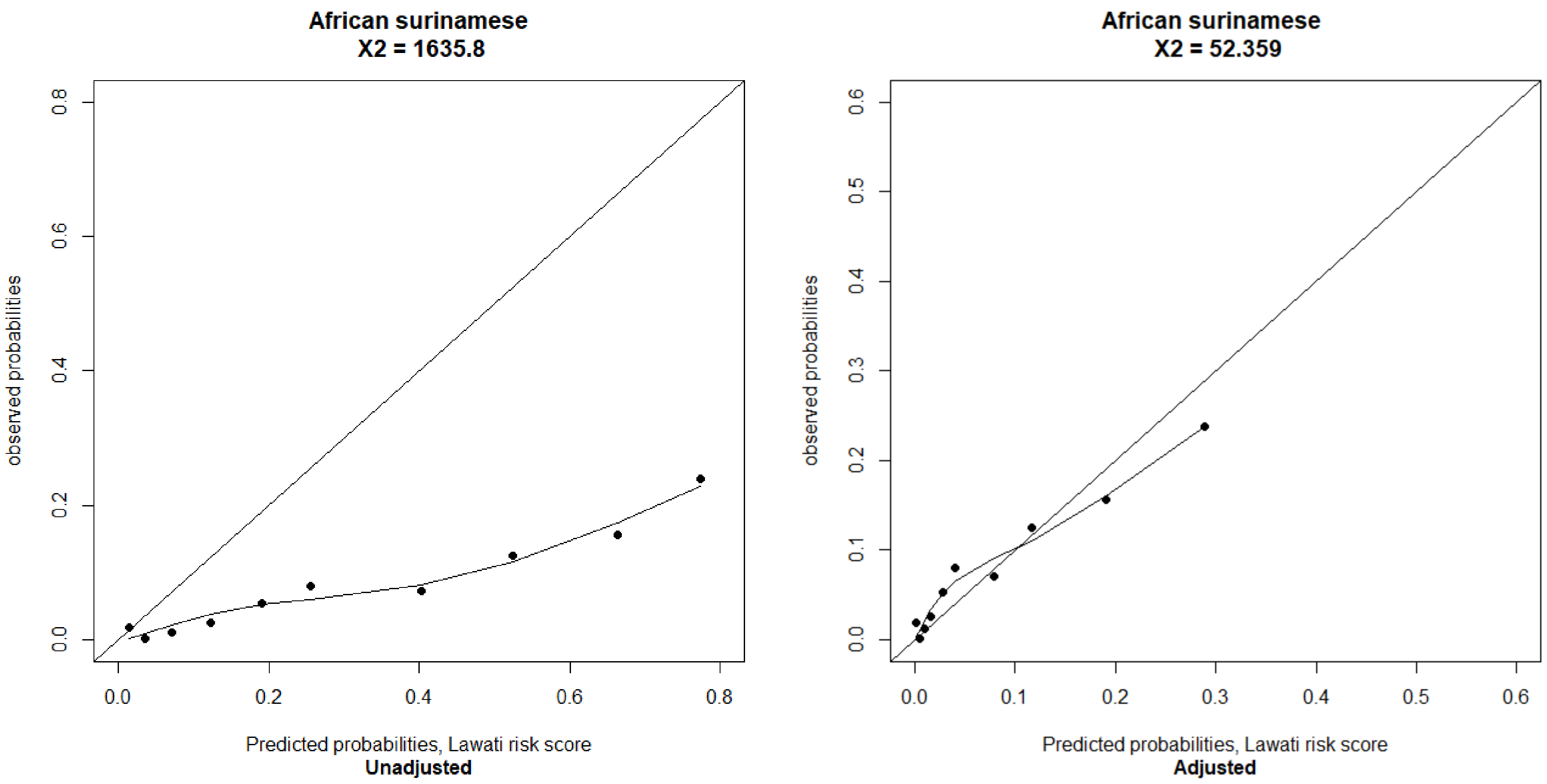
African surinamese
X2 = 1635.8
observed probabilities
0.0
0.2
0.4
0.6
0.8
Predicted probabilities, Lawati risk score
Unadjusted
African surinamese
X2 = 52.359
observed probabilities
0.0
0.1
0.2
0.3
0.4
0.5
0.6
Predicted probabilities, Lawati risk score
Adjusted

E.

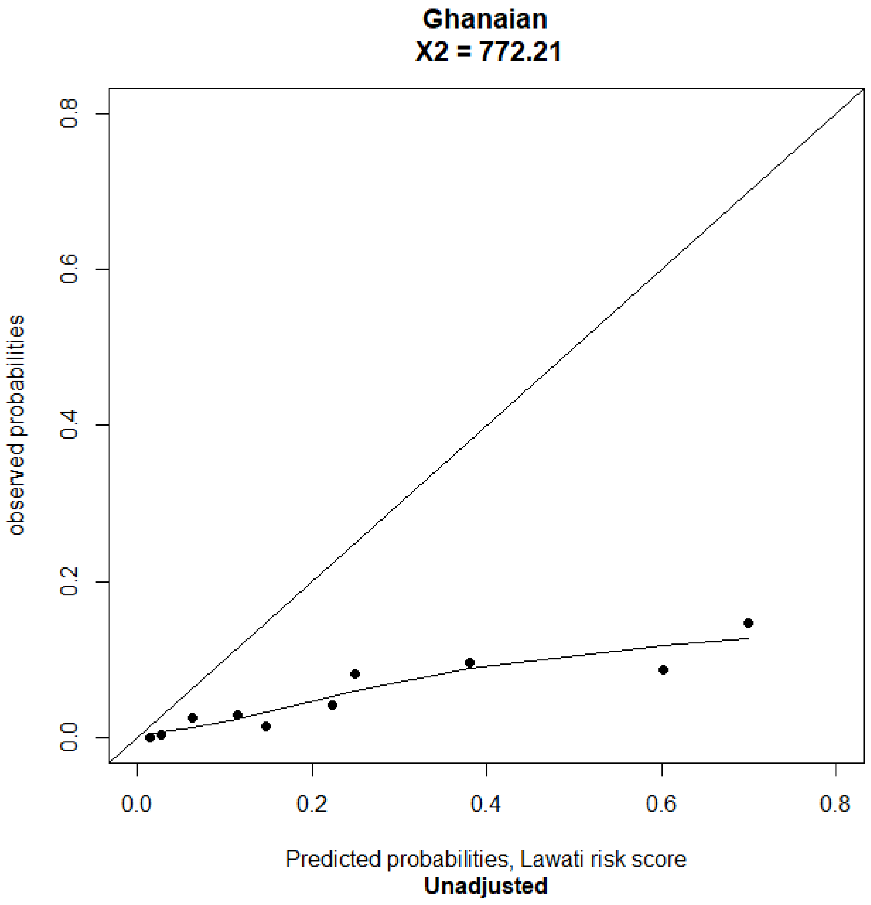

Ghanaian
X2 = 772.21
observed probabilities
0.0
0.2
0.4
0.6
0.8
Predicted probabilities, Lawati risk score
Unadjusted

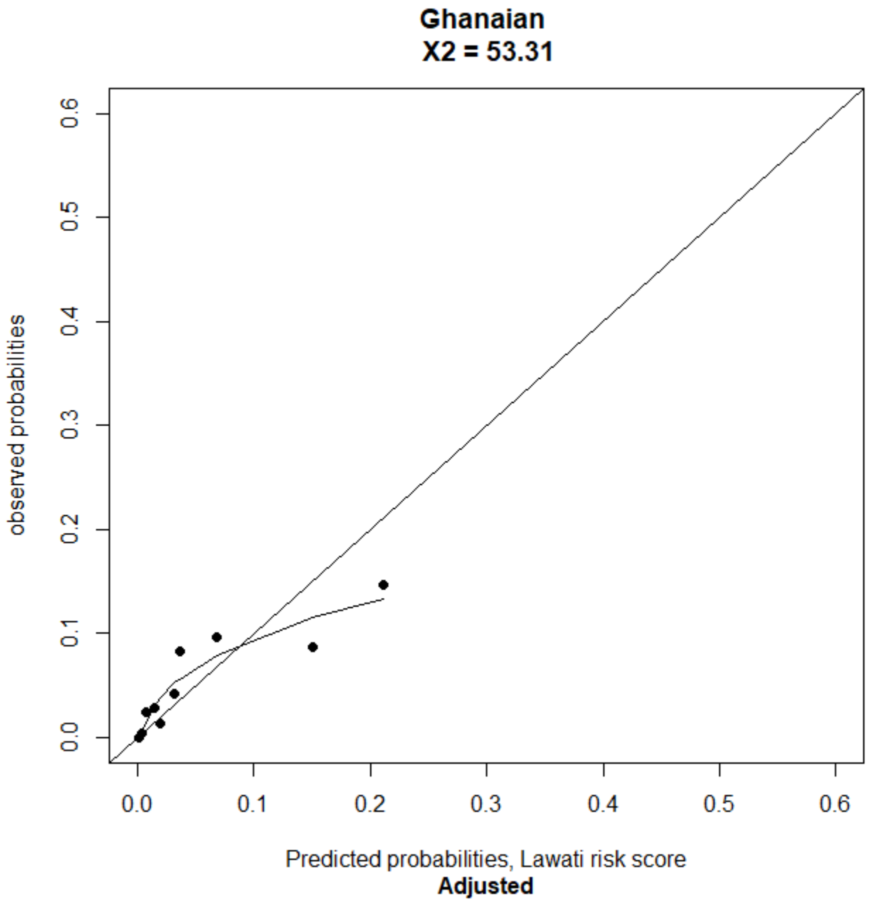

Ghanaian
X2 = 53.31
observed probabilities
0.0
0.1
0.2
0.3
0.4
0.5
0.6
Predicted probabilities, Lawati risk score
Adjusted

F.

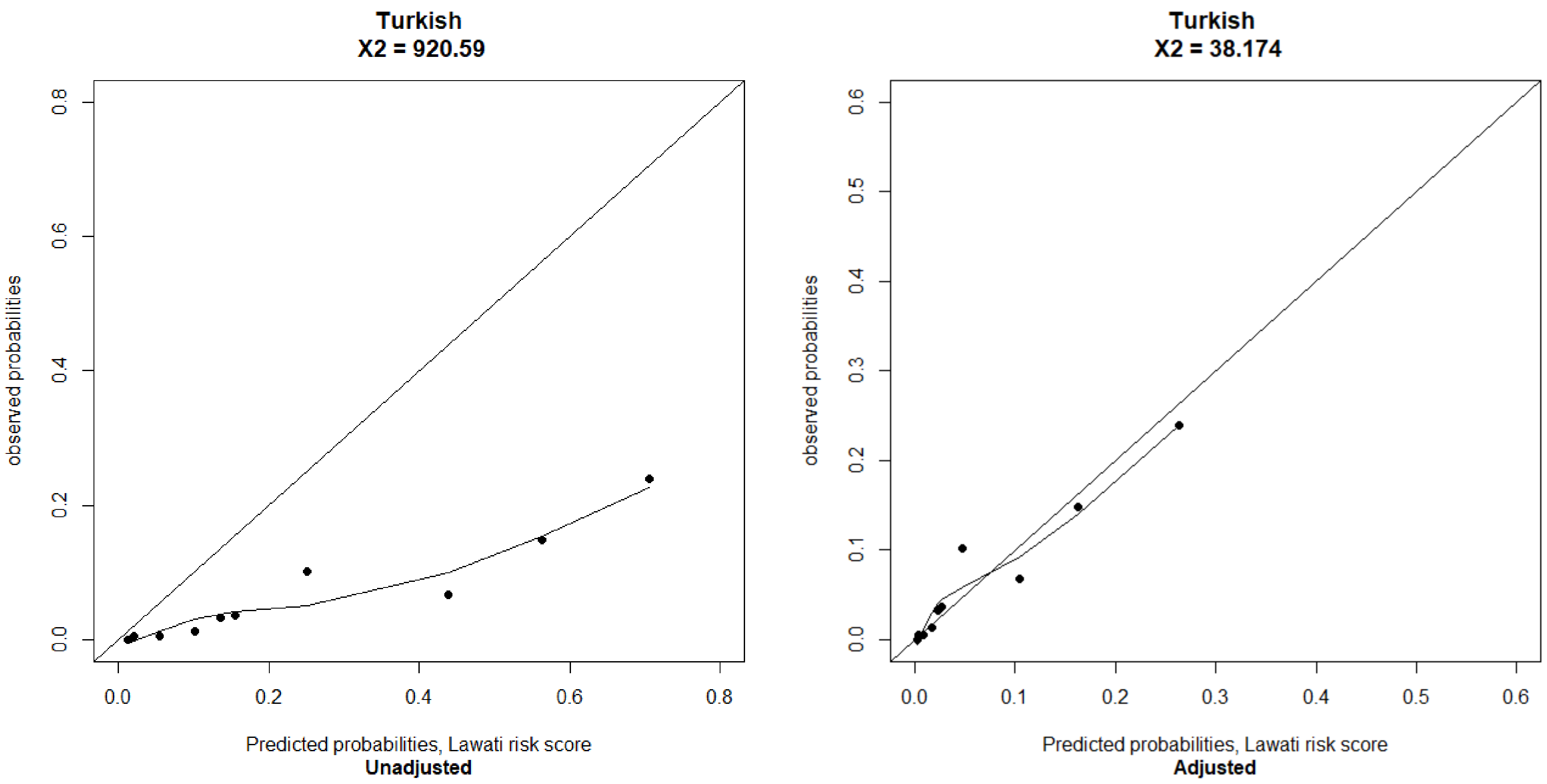
Turkish
X2 = 920.59
observed probabilities
0.0
0.2
0.4
0.6
0.8
Predicted probabilities, Lawati risk score
Unadjusted
Turkish
X2 = 38.174
observed probabilities
0.0
0.1
0.2
0.3
0.4
0.5
0.6
Predicted probabilities, Lawati risk score
Adjusted

G.

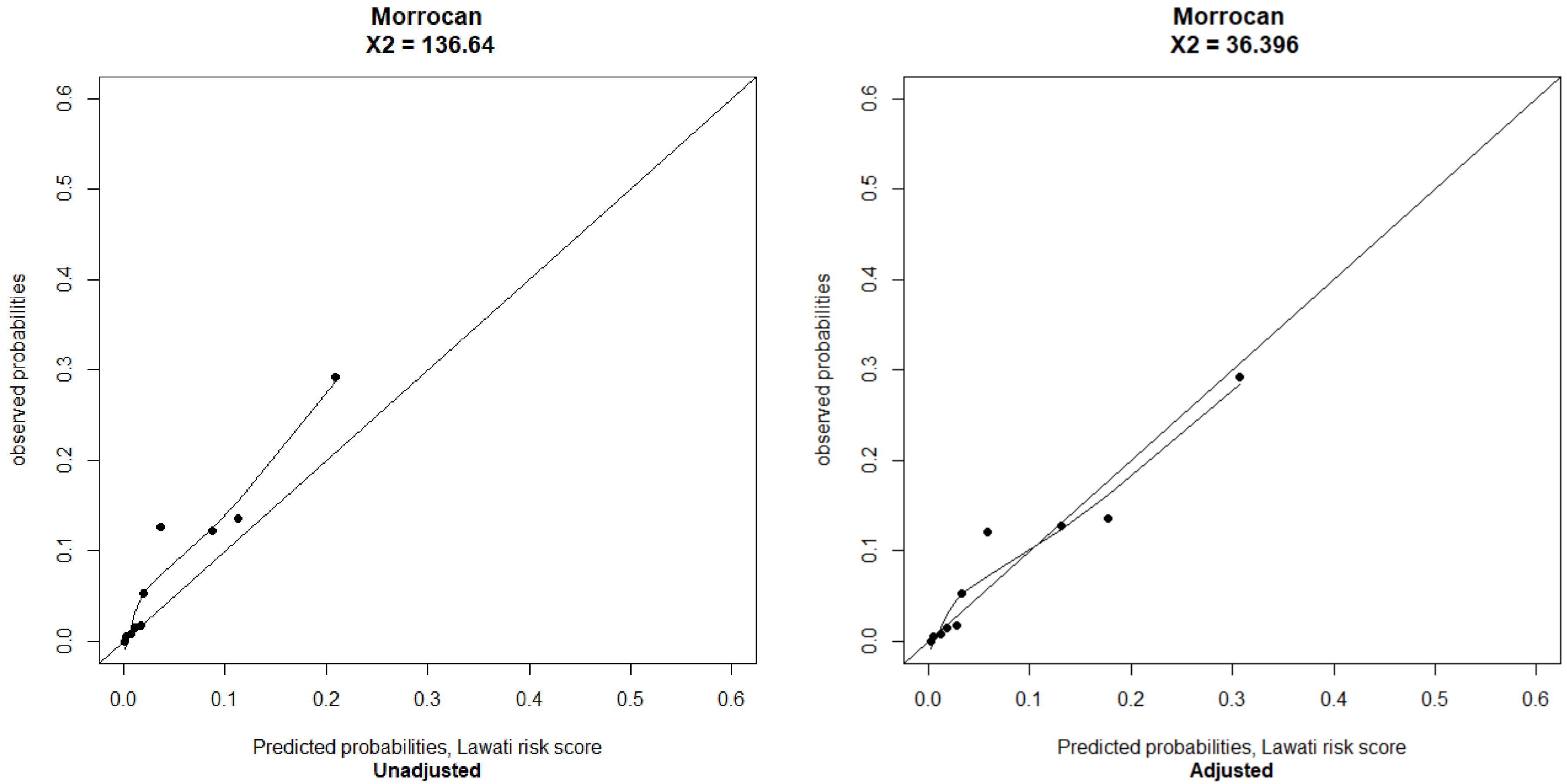

Supplementary fig 2: Calibration plots (including hosmer lemeshow's chi-square statistics) for adjusted and unadjusted Lawati et al's (1) risk assessment model per ethnic group (A. Total; B. Dutch; C. Asian Surinamese; D. African Surinamese; E. Ghanaian; F. Turkish and G. Moroccans). The plot depicts predicted risk against observed risk of having undiagnosed type 2 diabetes in our validation dataset.

REFERENCES

1. Al-Lawati JA, Tuomilehto J. Diabetes risk score in Oman: a tool to identify prevalent type 2 diabetes among Arabs of the Middle East. Diabetes Res Clin Pract. 2007;77(3):438-44.
